# Myasthenia Gravis Is Not an Independent Prognostic Factor of Thymoma: Results of a Propensity Score Matching Trial of 470 Patients

**DOI:** 10.3389/fonc.2020.583489

**Published:** 2020-11-27

**Authors:** Yirui Zhai, Yong Wei, Zhouguang Hui, Yushun Gao, Yang Luo, Zongmei Zhou, Qinfu Feng, Yuemin Li

**Affiliations:** ^1^ Department of Radiation Oncology, National Cancer Center/National Clinical Research Center for Cancer/Cancer Hospital, Chinese Academy of Medical Sciences & Peking Union Medical College, Beijing, China; ^2^ Department of Radiation Oncology, The 8th Medical Center of Chinese PLA General Hospital, Beijing, China; ^3^ Graduate School, Hebei North University, Zhangjiakou, China; ^4^ Department of VIP Medical Services, National Cancer Center/National Clinical Research Center for Cancer/Cancer Hospital, Chinese Academy of Medical Sciences & Peking Union Medical College, Beijing, China; ^5^ Department of Thoracic Surgery, National Cancer Center/National Clinical Research Center for Cancer/Cancer Hospital, Chinese Academy of Medical Sciences & Peking Union Medical College, Beijing, China; ^6^ Department of Medical Oncology, National Cancer Center/National Clinical Research Center for Cancer/Cancer Hospital, Chinese Academy of Medical Sciences & Peking Union Medical College, Beijing, China

**Keywords:** myasthenia gravis, thymoma, propensity score matching, survival, prognosis

## Abstract

**Objective:**

The association between the prognosis of thymoma and MG remains controversial. Differences in clinical characteristics and treatments between patients with and without MG may affect the findings of those studies. We designed this propensity score matching trial to investigate whether MG is an independent prognostic predictor in thymoma.

**Methods:**

Patients with pathologically diagnosed thymoma and MG were enrolled in the MG group. Moreover, the propensity score matching method was used to select patients who were diagnosed with thymoma without MG from the database of two participating centers. Matched factors included sex, age, Masaoka stage, pathological subtypes, and treatments. Matched patients were enrolled in the non-MG group. Chi-squared test was used to compare the characteristics of the two groups. Overall survival, local-regional relapse-free survival, distant metastasis-free survival, progression-free survival, and cancer-specific survival were calculated from the diagnosis of thymoma using the Kaplan–Meier method.

**Results:**

Between April 1992 and October 2018, 235 patients each were enrolled in the MG and non-MG groups (1:1 ratio). The median ages of patients in the MG and non-MG groups were 46 years old. The World Health Organization pathological subtypes were well balanced between the two groups (B2 + B3: MG vs. non-MG group, 63.0 vs. 63.4%, p = 0.924). Most patients in both groups had Masaoka stages I–III (MG vs. non-MG group, 90.2 vs. 91.5%, p = 0.631). R0 resections were performed in 86.8 and 90.2% of the MG and non-MG groups, respectively (p = 0.247). The median follow-up time of the two groups was 70.00 months (MG vs. non-MG group, 73.63 months vs. 68.00 months). Five-year overall survivals were 92.5 and 90.3%, 8-year overall survivals were 84.2 and 84.2%, and 10-year overall survivals were 80.2 and 81.4% (p = 0.632) in the MG and non-MG groups, respectively. No differences were found in the progression-free survival, distant metastasis-free survival, and local-regional relapse-free survival between the two groups.

**Conclusion:**

MG is not an independent or direct prognostic factor of thymoma, although it might be helpful in diagnosis thymoma at an early stage, leading indirectly to better prognosis.

## Introduction

Thymic epithelia neoplasm, including thymoma and thymic carcinoma, accounts for 0.2–1.5% of malignancies and is a rare disease with an incidence of 0.013–1.5 per million people (1, 2). Thymic carcinoma is typically characterized by more extensive local invasion, more frequent metastases, and a worse prognosis compared with thymoma. Paraneoplastic syndrome is relatively common in patients with thymoma. Although a wide range of such syndromes have been reported, including pure red blood cell aplasia, Good’s syndrome, and myasthenia gravis (MG), caused by impaired neuromuscular transmission resulting from the presence of antibodies at the neuromuscular junction is the most common paraneoplastic disease (3). Occurring in approximately 30–50% of patients with thymoma, MG is deemed a special characteristic (3, 4). Oppositely, the occurrence of MG is much lower in thymic carcinoma, at only 0–30%, probably because of the lack of thymus-like features compared with thymoma (5, 6). In patients with MG, thymoma has been found in approximately 8.5–15% of cases (7, 8). Thymoma is considered to be a negative prognostic factor of MG, (7) although whether MG is a prognostic factor of thymoma is still controversial (9–17). Differences in clinical characteristics and treatments between patients with and without MG may have affected the findings of previous studies (11, 12, 15, 17). From a clinical point of view, this controversy is important. Therefore, we designed this trial to investigate whether MG is an independent prognostic predictor in thymoma and used the propensity score matching method to eliminate the bias from other variables.

## Materials and Methods

### Ethics

The Institutional Ethics Committees of National Cancer Center/National Clinical Research Center for Cancer/Cancer Hospital, Chinese Academy of Medical Sciences & Peking Union Medical College and the 8th Medical Center of Chinese PLA General Hospital gave approval for the trial protocol, and informed written consent including the therapeutic regimens and possible data collections for the future academic analysis was obtained from patients before the treatment.

### Patient Selection

We searched for patients with pathologically diagnosed thymoma at the two medical centers between January 1992 and December 2018. Patients with pathologic diagnosis of thymoma and complete clinical information and follow-up data were identified. Thymic carcinoma was excluded. Data on sex, age, Masaoka stage, World Health Organization histological type, MG status, therapeutic regimens, and follow-up records were collected. Next, patients in the database were divided into two groups: MG and non-MG, according to their MG status. Then, propensity score matching (PSM) was used to achieve balance in clinicopathological characteristics.

### Statistical Analysis

In PSM, the matching ratio was 1:1 ratio, and the caliper is 0.05. Matched factors included sex, age, Masaoka stage, TNM stage (American Joint Committee on Cancer, 8th Edition), pathological subtypes, and treatments included surgery, radiotherapy, and chemotherapy. The chi-square test was used to compare characteristics of the two groups. Overall survival (OS), local-regional relapse-free survival (LRFS) distant metastasis-free survival (DMFS), progression-free survival (PFS), and cancer-specific survival (CSS) were calculated for based on the diagnosis of thymoma. OS was calculated from the time to death. LRFS was calculated as the time to local progression, which defined the mediastinal recurrence and supraclavicular lymph node relapse. DMFS was calculated as the time to distant metastasis. PFS was calculated as the time to documented clinical progression or to the patient’s death. CSS was calculated as the time to death from thymoma. All the survivals’ calculation utilized the Kaplan–Meier method. Univariate analysis was performed using the log-rank test. A p-value of <0.05 was considered statistically significant. PSM and other statistical analyses were performed using SAS software (Cary, NC, USA) and the SPSS statistical software package version 24.0 (SPSS Inc., Chicago, IL, USA), respectively.

## Results

### Patients’ Characteristics and Treatment

Overall, 927 patients were eligible to be enrolled in the database. Among them, 243 patients had MG. After PSM, 235 patients were enrolled into each group. There were 135 and 141 men in the non-MG and MG groups, respectively (p = 0.926). The median age was 46 years in both groups. The World Health Organization pathological subtypes were well balanced between the two groups (B2 + B3: non-MG vs. MG group, 63.4 vs. 63.0%, p = 0.924). Most patients in both groups had Masaoka stages I–II and TNM stages I–III (non-MG vs. MG group, 91.5 vs. 90.2%, p = 0.631).

R0 resections were performed in 212 and 204 patients in the non-MG and MG groups, respectively. Ninety-two and 42 patients in the non-MG group received radiotherapy and chemotherapy, respectively. The corresponding numbers of patients in the MG group were 102 and 29, respectively. The median radiation doses in the non-MG and MG groups were 50 Gy (40–60 Gy) and 50 Gy (20–60 Gy), respectively. The median chemotherapy cycles in the non-MG and MG groups were 2 (1–10) and 2 (1–6), respectively.

The clinicopathological variables were well balanced. Details are shown in **Table 1**.

**Table 1:** Patients’ Characteristics.

Number of patients (%)	Non-MG	MG	p-value
**Number**	235	235	
**Age (years)**	46 (17–84)	46 (16–78)	0.563
**≤50**	149 (63.4)	155 (66.0)	
**>50**	86 (36.6)	80 (34.0)	
**Sex**			0.574
**Male**	135 (57.4)	141 (60.0)	
**Female**	100 (42.6)	94 (40.0)	
**Masaoka stage**			0.631
**I**	119 (50.6)	103 (43.8)	
**II**	72 (30.6)	70 (29.8)	
**III**	24 (10.2)	39 (16.6)	
**IV**	20 (8.5)	23 (9.8)	
**Histology**			0.924
**A**	17 (7.2)	10 (4.3)	
**AB**	37 (15.7)	41 (17.4)	
**B1**	32 (13.6)	36 (15.3)	
**B2**	90 (38.3)	77 (32.7)	
**B3**	47 (20.0)	54 (23.0)	
**Mixed B2 and B3**	12 (5.1)	17 (7.2)	
**T stage**			0.385
**T1**	192 (81.7)	176 (74.9)	
**T2**	5 (2.1)	10 (4.3)	
**T3**	29 (12.3)	38 (16.2)	
**T4**	9 (3.8)	11 (4.7)	
**N stage**			0.890
**N0**	225 (95.7)	226 (96.2)	
**N1**	6 (2.6)	5 (2.1)	
**N2**	4 (1.7)	4 (1.7)	
**M stage**			0.945
**M0**	217(92.3)	216 (91.9)	
**M1a**	14 (6.0)	14 (6.0)	
**M1b**	4 (1.7)	5 (2.1)	
**TNM stage**			0.631
**I**	190 (80.6)	174 (74.0)	
**II**	5(2.1)	6 (2.6)	
**III**	20 (8.5)	32 (13.6)	
**IV**	20(8.5)	23 (9.8)	
**R0 resection**			0.247
**Yes**	212 (90.2)	204 (86.8)	
**No**	23 (9.8)	31 (13.2)	
**Radiation**			0.349
**Yes**	92 (39.1)	102 (43.4)	
**No**	143 (60.9)	133 (56.6)	
**Chemotherapy**			0.094
**Yes**	42 (17.9)	29 (12.3)	
**No**	193 (82.1)	206 (87.7)	

MG, myasthenia gravis; TNM, tumor, node, and metastasis.

Statistical significance is defined as p-value <0.05.

Values are presented as numbers (percentages).

### MG

In the MG group, MG symptoms were classified according to the Osserman classification. The number of patients with Types I, II, III, and IV were 85 (36.2%), 125 (55.2%), 16 (6.8%), and 9 (3.8%), respectively.

### Survival

**Figure 1:**
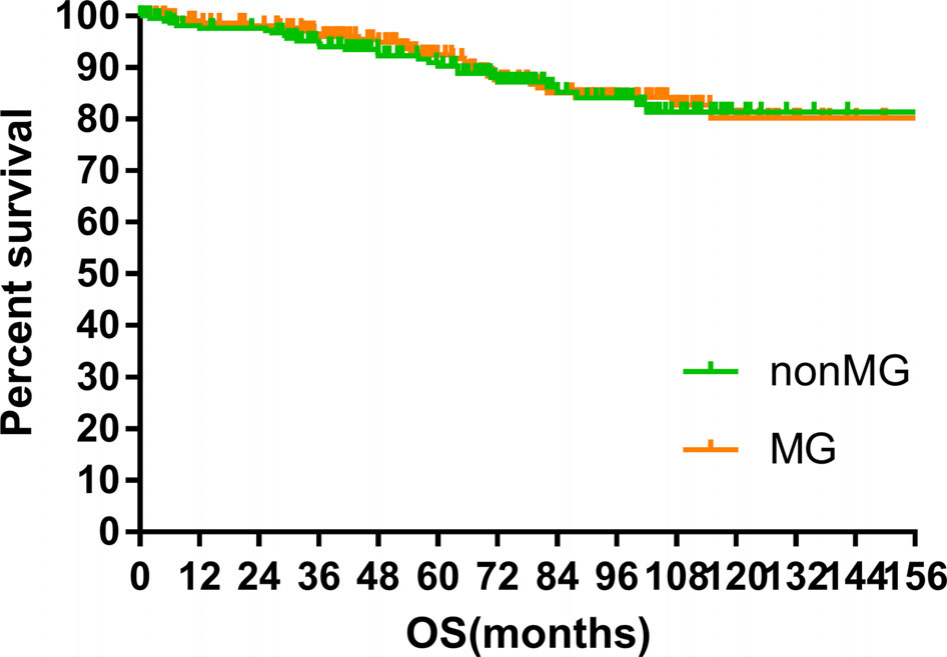
Overall survival.

**Figure 2:**
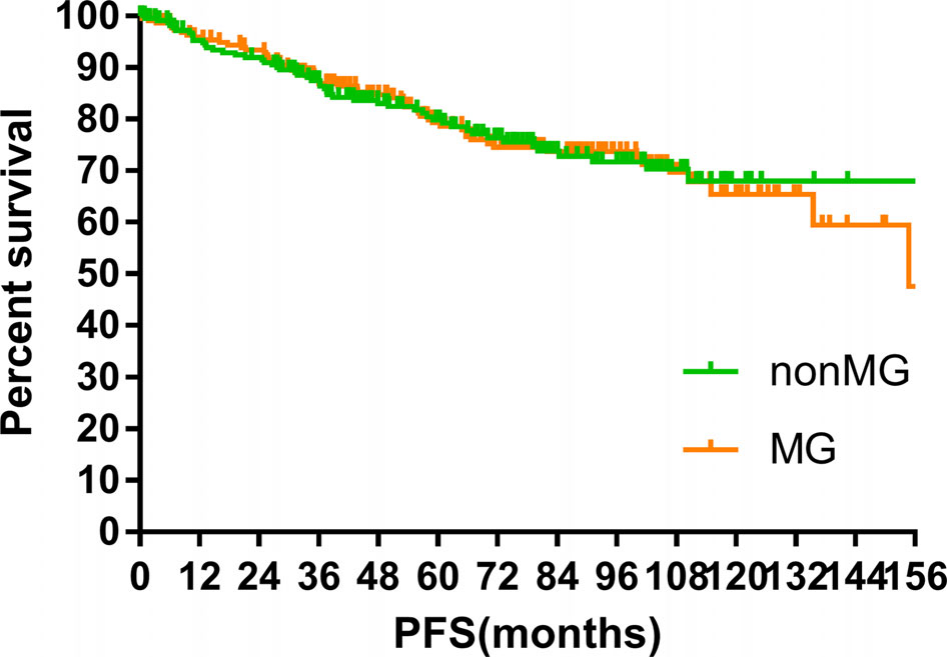
Progression-free survival.

**Figure 3:**
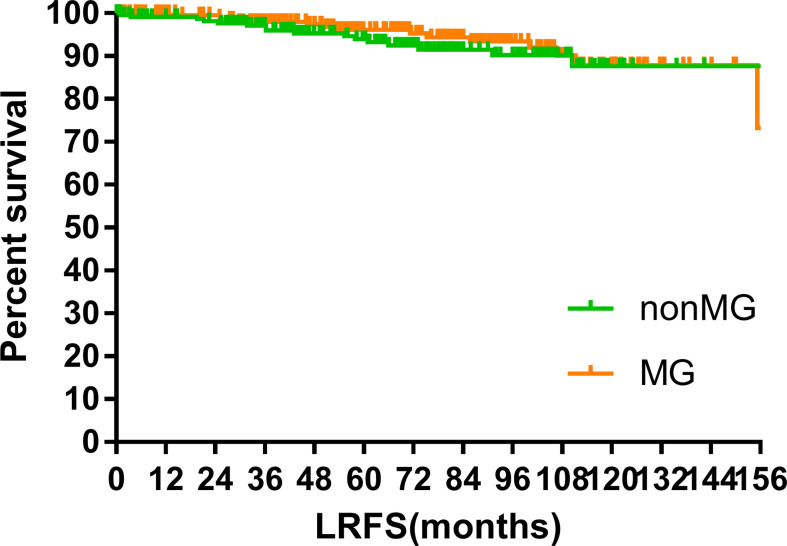
Local-regional relapse free survival.

**Figure 4:**
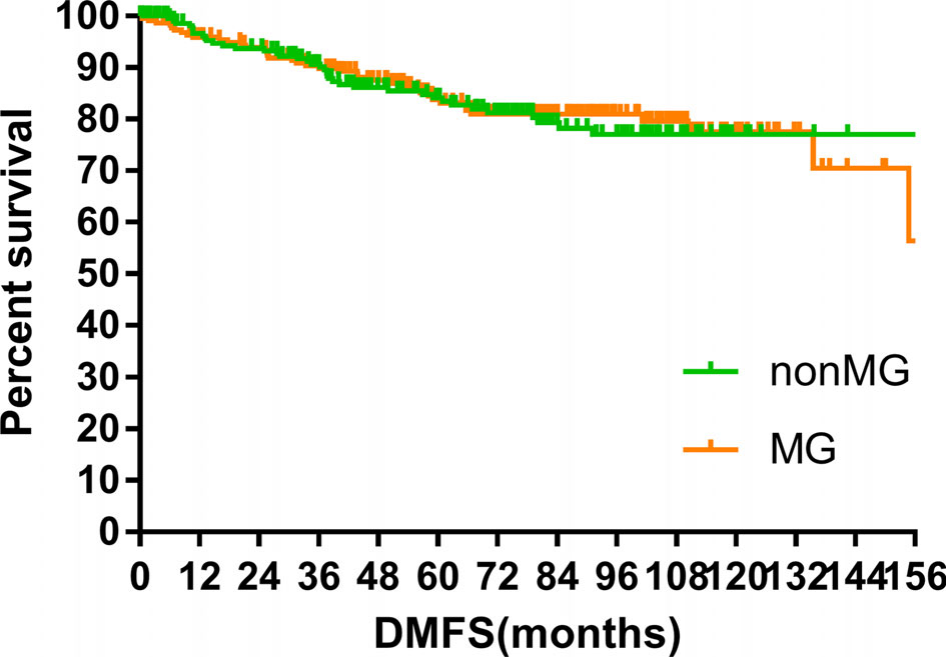
Distant metastasis free survival.

**Figure 5:**
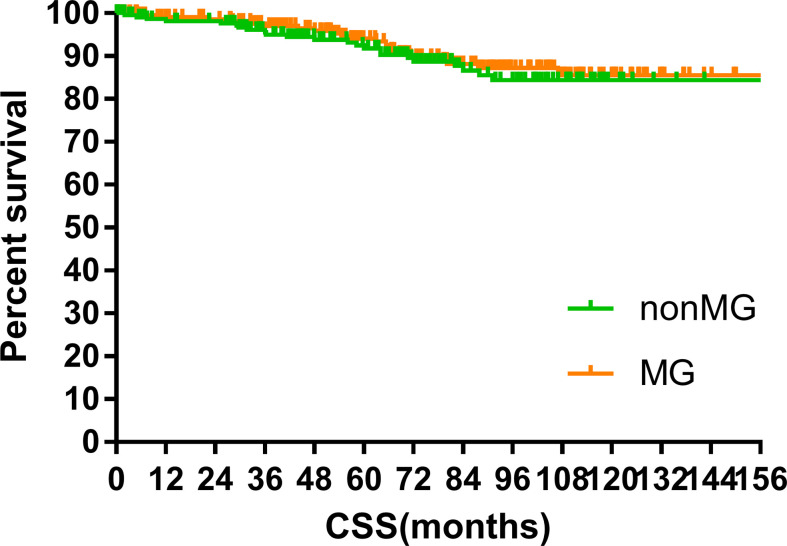
Cancer specific survival.

The median follow-up time of the two groups was 70.00 months (MG vs. non-MG group, 73.63 months vs. 68.00 months). At the last follow-up, 28 patients had died in each group. Five patients died of myasthenic crisis without progression of thymoma. Local-regional recurrences were observed in 15 and 16 patients in the MG and non-MG groups, respectively. Distant metastasis was observed in 39 patients in each group. The 5-year OS rates were 90.3 and 92.5%, 8-year OS rates were 84.2 and 84.2%, and 10-year OS rates were 81.4 and 80.2% (p = 0.632) in the non-MG and MG groups, respectively. The respective PFS rates were 79.8 and 79.3%, 71.7 and 73.7%, and 67.9 and 65.4% (p = 0.832) in the non-MG and MG groups. The two groups also had similar 5-year (93.9 vs. 96.1%), 8-year (90.2 vs. 93.4%), and 10-year LRFS rates (87.7 vs. 87.8%, p = 0.613). Additionally, the 5-, 8-, and 10-year DMFS rates in the non-MG group were almost equal to those in the MG group (84.1 vs. 83.8%, 77.1 vs. 81.0%, 77.1 vs. 77.5%, p = 0.884). There were no significant differences in the 5-, 8-, and 10-year CSS rates between the two groups (91.8 vs. 94.0%, 84.4 vs. 87.2%, 84.4 vs. 85.6%, p = 0.441). The survival curves are plotted in **Figures 1**–**5**.

### Subgroup Analyses

Subgroup analysis according to Masaoka stage and TNM stage did not show any significant differences in OS, LRFS, DMFS, CSS, or PFS between patients with or without MG. However, patients with stage IV (both TNM and Masaoka) in the MG group had somewhat better OS than those in the non-MG group. Details are shown in **Table 2**.

**Table 2:** Subgroup Analyses.

Survival	Masaoka stage	Non-MG%	MG%	p-value	TNMstage	Non-MG%	MG%	p-value
5y OS	I	94.8	93.7	0.299	I	93.8	94.1	0.625
	II	91.7	94.7	0.630	II	100.0	100.0	–^*^
	III	89.4	93.9	0.820	III	87.4	92.5	0.949
	IV	57.5	76.4	0.069	IV	57.5	76.4	0.069
5y PFS	I	95.0	89.9	0.125	I	89.5	91.0	0.815
	II	79.4	92.4	0.166	II	66.7	88.3	0.127
	III	46.4	57.1	0.105	III	43.2	50.9	0.217
	IV	33.7	33.5	0.875	IV	33.7	33.5	0.875
5y LRFS	I	98.0	98.9	0.850	I	95.4	99.3	0.732
	II	90.6	100.0	0.461	II	100.0	100.0	–^*^
	III	94.1	87.6	0.388	III	92.9	84.5	0.504
	IV	77.0	88.2	0.593	IV	77.0	88.2	0.593
5y DMFS	I	96.7	95.1	0.782	I	92.4	94.7	0.343
	II	84.5	94.0	0.088	II	66.7	88.3	0.127
	III	46.4	63.3	0.190	III	43.2	58.3	0.313
	IV	52.1	38.7	0.731	IV	52.1	38.7	0.731
5y CSS	I	95.7	96.2	0.609	I	94.9	95.6	0.929
	II	93.2	94.7	0.631	II	100.0	100.0	–^*^
	III	89.4	96.6	0.628	III	87.4	95.7	0.751
	IV	63.9	76.4	0.133	IV	63.9	76.4	0.133

^*^P value is not defined because of limited number of patients in each group. MG, myasthenia gravis; OS, overall survival; PFS, progression-free survival; LRFS, local-regional relapse-free survival; DMFS, distant metastasis-free survival; CSS, cancer-specific survival.

Statistical significance is defined as p-value <0.05.

Values are presented as percentages.

## Discussion

Thymoma, which accounts for 20% of mediastinal neoplasm, is the most frequent primary malignancy. MG is deemed as a special characteristic of thymoma compared with other thoracic malignancies (1, 4, 8). In previous reports, 5- and 10-year OS rates were 63.9–90.0% and 70.9–82.0%, for patients without MG, respectively. Five- and 10-year OS rates were 76.0–93.6% and 62.0–83.0% for patients without MG (7, 9, 10, 12–17). The results in our study are consistent with those in previous studies.

Whether MG influences the prognosis of thymomas has long been controversial. In the 1970s, MG was identified as a negative prognostic factor of thymoma according to the poor experience and therapeutic regimens with MG and MG-related postoperative complications (18). Once the therapeutic technologies for MG progressed, the role of MG in the prognosis of thymoma took a favorable turn. Recently, only one study identified MG as a negative indicator of prognosis, and that study had a severe bias of radiation delivery between the two groups (17). Other studies came to the opposite conclusion. Recent studies are listed in **Table 3**. A study by Wang et al. showed that patients with MG had higher OS than those without MG in univariate analysis. However, this advantage disappeared in multi-variate analysis (15). Another study by Ruffini et al. showed similar results as that in Wang et al.’s (13). Two other studies by Filosso et al. and Kondo et al. did not show statistically significant differences in OS between patients with and without MG, although patients with MG had better OS (11, 12). Interestingly, the slight difference in OS disappeared at the 10th year in the study by Filosso et al. (11). Results from the other four studies showed no significant differences (9, 10, 14, 16). We analyzed the characteristics of the patients enrolled in previous studies and found that in none of the characteristics of the patients were well balanced. In a study by Ruffini et al., there is an obvious correspondence between MG and other features that might contribute to the effect on prognosis (13). Generally, patients in the MG group always had a higher proportion of favorable prognostic factors, including Masaoka stages I–II, AB/B1 classification, and R0 resection (12, 15, 17). The study designed by Wang et al., which showed the most significant differences in survival measures, also showed the most imbalances in the characteristics of the patients (15). Additionally, we found that more the imbalances, more likely was the study to show differences in survival. For example, imbalances existed in six aspects (sex, age, histology, R0 resection, chemotherapy, and radiotherapy) in the study by Wang et al. (15). Previous studies showed that these factors obviously influence survival. The studies by Filosso et al. and Kondo et al. showed four and three imbalanced factors, respectively. There was only one imbalanced factor in the other studies, which showed no significant differences in survival (9, 10, 14, 16). In an attempt to reduce the imbalances, four of the studies had conducted multivariate analysis, and none of them demonstrated the effect of MG on survival (11, 13, 15, 16). Our study, utilizing the PSM method, also tried to balance other substantially influencing factors, and we found no difference in survival measures between patients with and without MG. From the historical studies and our study, one can reach a conclusion that once the other influencing factors were well balanced, MG appeared not to be an independent positive indicator in thymoma. The positive prognostic effect of MG in previous studies is probably the result of patients with MG being more eager for precise diagnosis at an earlier stage, with the disease histologically expressed as less invasive.

**Table 3:** Survival Measures in Previous Studies.

	Year	Number of patients Non-MG vs. MG	Imbalanced characteristics	OS Non-MG vs. MG	p-value
Cacho-Díaz et al. (10)	2018	46 vs. 18	WHO stage	MST 120.6 m vs. NR	0.606
Zhang et al. (17)	2016	66 vs. 38	Age, histology, Masaoka stage, radiotherapy	5y 89.1 vs. 76.0%	0.026
Wang et al. (15)	2016	1429 vs. 421	Sex, age, histology, R0 resection, chemotherapy, radiotherapy.	5y 88 vs. 93% 10y 81 vs. 83%	0.034^a^ 0.967^b^
Aydemir (9)	2016	34 vs. 24	histology	5y 82.4 vs. 87.5%	0.311
Filosso et al. (11)	2015	422 vs. 375	Sex, stage, histology, induction therapy.	5y 84.9 vs. 93.6% 10y 70.9 vs. 77.2%	0.058^a^ 0.956^b^
Yu et al. (16)	2012	103 vs. 125	Histology	5y 90 vs. 89.3% 10y 78.9 vs. 81.2%	0.886^b^
Vachlas et al. (14)	2012	40 vs. 39	Histology	MST 15.7y vs. 14.5y	0.681
Ruffini et al. (13)	2011	150 vs. 105	Masaoka stage, histology	10y 62 vs. 82%	0.001^a^ 0.88^b^
Kondo et al. (12)	2005	770 vs. 259	Age, histology, resection,	5y 89.3 vs. 85.7%^c^ 5y 63.9 vs. 85.1%^d^	>0.05^c^ 0.052^d^

^a^univariate analysis, ^b^multivariate analysis, ^c^stage III, ^d^stage IV.

MG, myasthenia gravis; MST, median survival time; NR, not reached; OS, overall survival; WHO, World Health Organization; y, year.

Statistical significance is defined as p-value <0.05.

Values are presented as numbers (percentages).

Several studies insisted that the effect of MG on survival was according to the Masaoka stage. The views of the effect of MG on prognosis differed quite dramatically among studies. In the study by Ruffini et al., difference in OS was observed only in patients with Masaoka stage I (10-year OS in nonMG and MG groups: 80 vs.100%, p = 0.02) (13). Wang et al. showed that the survival rate was significantly higher in the non-MG group than in the MG group when the Masaoka staging was I (p = 0.000), equal when the Masaoka staging was II (p = 0.484), and significantly lower when the Masaoka staging was III/IV (P = 0.003) (15). The study by Kondo et al. clarified that although MG was not associated with survival in patients with stage III, OS tended to be better in patients with stage IV with MG than in those without MG (12). The results of subgroup analysis in our study were consistent with those of Kondo et al. The reason for this might be that thymoma is a disease with relatively better prognosis than other common thoracic malignancies and the causes of deaths in patients with thymoma are very complex. In long-term follow-up, patients might have other diseases or accidents. Therefore, CSS is a more representative measure to evaluate survival. However, few studies have focused on CSS, and our study did not show that MG is associated with CSS in any Masaoka stage.

Very few studies focused on MG as a prognostic factor of disease progression including local relapse and distant metastasis. A study from Italy evaluated whether MG is an indicator of the cumulative incidence of recurrence. It showed that 5- and 10-year progression rates were 10.7 and 14.7% in MG patients and 11.1 and 15.7% in non-MG patients (11). The recurrence rate in our study was consistent with this study. The study by Kondo et al. showed no recurrent difference between patients with and without MG (6.4 vs. 8.3%) (12). However, the frequency of recurrence in the non-MG group was higher than that in the MG group in stage IV (12). The study by Wang et al. showed higher recurrence rate in the non-MG group, although again, we cannot overlook the severe imbalances of other factors in that study (15). After using PSM to stabilize other factors, our study showed similar local recurrence, distant metastasis, and progression rates between the two groups.

Because this is the first study using PSM to investigate the relationship of MG and survivals of thymomas, our study has several strengths. First, because it is difficult to propose a prospective study in a rare disease, PSM is a stable substitutive method. The other influencing factors were reduced by using PSM, and it helped us to clearly see the effect of MG on prognosis of thymoma. Second, the number of patients was relatively large although thymoma is a rare disease. Third, the enrolled patients were all from two high-volume institutions, and the data are reliable. Last, the results are consistent with those of previous studies in which other clinical features were statistically analyzed using multivariate Cox regression models.

There are also some limitations to our study. First, because this was a retrospective study, treatments for thymoma and MG were not always the same. Second, the two participating centers have different academic advantages. One is a cancer center and the other is famous for treatments for MG. Inevitably, the treatment details are not totally consistent. Third, we made an effort to shrink the imbalances using PSM, although it is not realistic to achieve a perfect balance because the proportions of clinical features had their own peculiarity in the database before PSM, and there were minor differences in delivery of chemotherapy between the two groups.

In conclusion, MG is not an independent or direct prognostic factor of thymoma, although it might be helpful to get the diagnosis at an early stage, indirectly leading to better prognosis.

## Data Availability Statement

The raw data supporting the conclusions of this article will be made available by the authors, without undue reservation.

## Ethics Statement

The studies involving human participants were reviewed and approved by The Ethics Committees of National Cancer Center and the 8th Medical Center of Chinese PLA General Hospital. The patients/participants provided their written informed consent to participate in this study.

## Author Contributions

YZ, YW, YLi, and QF designed the study and wrote and reviewed the manuscript. YZ and YW collected the data, finished statistical analysis, and wrote the manuscript. ZH, YG, YLu and ZZ enrolled patients, collected the data, finished data interpretation and manuscript editing. All authors contributed to the article and approved the submitted version.

## Funding

This work was supported by PUMC FUND of the Funds for the Central Universities (No. 3332018075), CAMS Innovation Fund for Medical Sciences (No. 2019-I2M-2-003), and the National Key Projects of Research and Development of China (2016YFC 0904600).

## Conflict of Interest

The authors declare that the research was conducted in the absence of any commercial or financial relationships that could be construed as a potential conflict of interest.
